# Supplementary material for: The Effect of Crataegus Fruit Pre-Treatment and Preservation Methods on the Extractability of Aroma Compounds during Liqueur Production
Source: Molecules. 2022 Feb 23;27(5):1516. doi: 10.3390/molecules27051516 (PMC8911551; doi:10.3390/molecules27051516)
Supplement: Supplementary file 1 [file molecules-27-01516-s001.zip › molecules-1543539-supplementary.pdf]

Table S1. The least square means of the content of volatile compounds (g·100 g^{-1}) with the descriptions of the odors and classification into chemical groups as affected by the sucrose saturation and the preservation method applied to the hawthorn pseudo-fruits during liqueur preparation (Mean ± SE).

Name of the volatile compounds	Odor descriptor	Saturated with sucrose		Methods of preservation			
		Yes	No	Fresh	Frozen	Freeze-dried	Hot air-dried
**Acetals**							
acetaldehyde diethyl acetal	ethereal, green nut, earthy sweet, vegetable[1*]	6.320 ± 0.845 A	10.616 ± 3.288 B	2.773 ± 0.741 a	12.546 ± 3.751 b	3.919 ± 0.814 a	14.635 ± 2.954 b
isobutyraldehyde diethyl acetal		1.500 ± 0.250 B	0.799 ± 0.188 A	1.161 ± 0.422 ab	1.414 ± 0.136 b	1.191 ± 0.548 ab	0.832 ± 0.257 a
2-methylbutyraldehyde diethyl acetal		1.754 ± 0.184 A	8.208 ± 3.129 B	1.292 ± 0.278 a	12.052 ± 5.959c	3.210 ± 0.605 b	3.370 ± 0.818 b
3-methylbutyraldehyde diethyl acetal		3.935 ± 0.454 B	2.011 ± 0.396 A	4.010 ± 0.420 c	3.108 ± 0.725 b	2.723 ± 0.242 b	2.051 ± 0.118 a
**Alcohols**							
2-methyl-2-propanol	camphor[1*]	0.018 ± 0.003 A	0.029 ± 0.009 B	0.009 ± 0.000 a	0.014 ± 0.008 a	0.036 ± 0.014 b	0.036 ± 0.007 b
1-butanol	fusel oil, sweet, balsam, whiskey[1*]	0.105 ± 0.056 B	0.087 ± 0.010 A	0.210 ± 0.087 c	0.064 ± 0.038 b	0.041 ± 0.024 a	0.069 ± 0.004 b
hexanol	herbal[2*]	0.060 ± 0.022 A	0.066 ± 0.016 A	0.02 ± 0.013 a	0.081 ± 0.026 b	0.030 ± 0.006 a	0.121 ± 0.022 c
2-ethylhexanol	citrus[2*]	0.156 ± 0.067 B	0.110 ± 0.019 A	0.297 ± 0.087 d	0.136 ± 0.014 c	0.016 ± 0.009 a	0.084 ± 0.029 b
benzyl alcohol	floral[2*]	0.068 ± 0.016 A	0.116 ± 0.016 B	0.129 ± 0.006 c	0.101 ± 0.029 b	0.103 ± 0.025 b	0.035±0.007 a
2-hexyloctanol	waxy[2*]	9.614 ± 0.953 A	8.679 ± 0.649 A	10.134 ± 1.810 a	8.884 ± 0.475 a	9.062 ± 0.666 a	8.306±1.427 a
**Aldehydes**							
3-methylbutanal	aldehydic[2*]	0.372 ± 0.095 A	0.398 ± 0.021 B	1.031 ± 0.195 d	0.317 ± 0.113 c	0.140 ± 0.063 b	0.052 ± 0.010 a
2-methylbutanal	chocolate[2*]	0.263 ± 0.067 B	0.058 ± 0.004 A	0.141 ± 0.082 b	0.220 ± 0.056 bc	0.256 ± 0.014 c	0.023 ± 0.002 a
benzaldehyde	fruity, woody, burnt sugar[2,3*]	0.424 ± 0.047 A	0.729 ± 0.129 B	0.775 ± 0.259 b	0.661 ± 0.132 b	0.452 ± 0.053 a	0.418 ± 0.038 a
3-(2- or 4-) methylbenzaldehyde		0.299 ± 0.035 A	0.631 ± 0.075 B	0.633 ± 0.183 b	0.344 ± 0.079 a	0.396 ± 0.075 a	0.488 ± 0.085 ab
**Alkanes**							
5-propyldecane		2.667 ± 0.262 A	2.558 ± 0.190 A	2.713 ± 0.504 a	2.789 ± 0.095 a	2.781 ± 0.117 a	2.168 ± 0.358 a

4-methyltetradecane		0.720 ± 0.074 A	1.096 ± 0.263 B	0.500 ± 0.018 a	1.367 ± 0.413 c	0.887 ± 0.046 b	0.879 ± 0.257 b
**Alkenes**							
2,4-dimethyl-1-heptene		0.551 ± 0.017 B	0.172 ± 0.028 A	0.706 ± 0.031 d	0.439 ± 0.014 c	0.234 ± 0.016 b	0.067 ± 0.004 a
2,4-dimethyl-1-decene		3.152 ± 0.738 B	2.145 ± 0.344 A	4.257 ± 0.126 b	2.063 ± 0.556 a	2.158 ± 0.212 a	2.117 ± 0.574 a
4-methyl-1-undecene		1.386 ± 0.243 A	1.139 ± 0.084 A	1.864 ± 0.320 b	1.163 ± 0.093 a	1.092 ± 0.143 a	0.928 ± 0.161 a
**Aromatic hydrocarbons**							
toluene	pungent, caramel, ethereal, synthetic, fruity, rubbery,solvent-like[3*]	0.259 ± 0.050 A	0.356 ± 0.072 A	0.115 ± 0.023 a	0.356 ± 0.114 b	0.372 ± 0.079 b	0.389 ± 0.049 b
ethylbenzene	ethereal, floral, sweet[3*]	0.128 ± 0.038 A	0.269 ± 0.074 B	0.041 ± 0.002 a	0.172 ± 0.012 b	0.423 ± 0.091 c	0.159 ± 0.063 b
p-xylene	plastic, cold meat fat-like[3*]	0.464 ± 0.090 A	0.793 ± 0.219 B	0.210 ± 0.042 a	0.563 ± 0.013 b	0.631 ± 0.145 b	1.111 ± 0.346 c
styrene	balsamic[2*]	0.286 ± 0.093 A	2.284 ± 0.110 B	0.073 ± 0.020 a	3.680 ± 0.209 c	0.647 ± 0.086 b	0.740 ± 0.139 b
o-xylene	geranium[1*]	0.198 ± 0.034 A	0.760 ± 0.020 B	0.066 ± 0.007 a	0.838 ± 0.032 c	0.353 ± 0.089 b	0.659 ± 0.261 c
p- (*o*-) cymene	citrus, solvent[4*]	0.150 ± 0.034 A	0.256 ± 0.016 B	0.086 ± 0.004 ab	0.176 ± 0.025 b	0.496 ± 0.014 c	0.055 ± 0.013 a
**Esters-MUFA**							
butanedioic acid diethyl ester	mild fruity, cooked apple, ylang[1*]	0.079 ± 0.015 B	0.030 ± 0.011 A	0.084 ± 0.018 b	0.012 ± 0.004 a	0.048 ± 0.019 ab	0.074 ± 0.003 b
diethyl malonate	sweet, fruity, green apple[1*]	0.032 ± 0.007 A	0.042 ± 0.007 A	0.032± 0.002 a	0.039 ± 0.009 a	0.044 ± 0.010 a	0.034 ± 0.004 a
diethyl methylsuccinate		0.078 ± 0.014 A	0.094 ± 0.021 A	0.088 ± 0.015 b	0.026 ± 0.002 a	0.134 ± 0.014 c	0.095 ± 0.019 b
ethyl-9-hexadecenoate		0.217 ± 0.015 A	0.175 ± 0.054 A	0.318 ± 0.041 c	0.098 ± 0.006 a	0.191 ± 0.005 b	0.178 ± 0.040 b
**Esters-PUFA**							
9,12,15-octadecatrienoic acid, ethyl ester		5.358 ± 0.894 B	3.383 ± 0.826 A	5.926 ± 0.822 b	1.973 ± 0.103 a	3.549 ± 0.039 a	6.036 ± 0.158 b
9,12-octadecadienoic acid, ethyl ester		3.348 ± 0.503 A	2.330 ± 0.601 A	3.995 ± 0.659 c	1.499 ± 0.073 a	2.344 ± 0.028 ab	3.518 ± 0.094b c
**Esters-SAFA**							
ethyl pentadecanoate	honey, sweet[1*]	0.547 ± 0.027 A	0.489 ± 0.055 A	0.585 ± 0.058 b	0.413 ± 0.061 a	0.538 ± 0.014 b	0.534 ± 0.014 b

ethyl acetate	ethereal, fruity, sweet, weedy green[1*]	3.182 ± 0.496 A	3.454 ± 0.485 A	1.903 ± 0.433 a	2.706 ± 0.310 a	4.239 ± 0.513 b	4.425 ± 0.493 b
ethyl butyrate	fruity juicy, fruit pineapple, cognac[1*]	0.184 ± 0.034 A	0.828 ± 0.403 B	0.110 ± 0.015 a	1.388 ± 0.073 b	0.322 ± 0.091 a	0.204 ± 0.044 a
ethyl decanoate	sweet, waxy, fruity, apple, grape oily, brandy[1,2*]	1.220 ± 0.128 B	0.898 ± 0.242 A	1.008 ± 0.054 b	0.984 ± 0.052 ab	1.414 ± 0.207 c	0.920 ± 0.111 a
ethyl dodecanoate	sweet, waxy, soapy and rummy with a creamy, floral nuance[1*]	9.192 ± 0.685 B	6.286 ± 0.129 A	4.454 ± 0.979 a	8.091 ± 0.117 b	10.820 ± 0.601 c	7.591 ± 0.181 b
ethyl heptadecanoate		0.242 ± 0.023 B	0.150 ± 0.028 A	0.212 ± 0.007 b	0.127 ± 0.045 a	0.229 ± 0.009 b	0.215 ± 0.068 b
ethyl hexadecanoate	waxy, fruity, creamy and milky with a balsamic nuance[1,3*]	35.016 ± 1.096 A	29.774 ± 5.810 A	40.075 ± 5.091 b	20.114 ± 7.705 a	35.875 ± 2.301 b	33.514 ± 2.704 b
ethyl hexanoate	sweet, fruity pineapple, waxy, green banana[1*]	0.267 ± 0.066 A	0.434 ± 0.085 B	0.183 ± 0.069 a	0.240 ± 0.012 ab	0.313 ± 0.059 b	0.667 ± 0.089 c
ethyl octanoate	fruity, wine, waxy, sweet, apricot, banana, brandy, pear[1,2*]	1.620 ± 0.473 A	1.984 ± 0.621 A	2.615 ± 0.961 c	2.059 ± 0.099 b	1.796 ± 0.588 b	0.739 ± 0.084 a
ethyl stearate	mild waxy[1*]	0.772 ± 0.139 B	0.500 ± 0.142 A	0.811 ± 0.149 c	0.271 ± 0.016 a	0.554 ± 0.020 b	0.909 ± 0.028 c
ethyl tetradecanoate	sweet, waxy[1,2*]	4.149 ± 0.160 B	1.384 ± 0.152 A	2.643 ± 0.064 ab	2.338 ± 0.089 a	3.283 ± 0.082 c	2.802 ± 0.085 b
ethyl undecanoate	soapy, waxy, fatty, cognac, coconut[1*]	0.080 ± 0.002 B	0.057 ± 0.002 A	0.106 ± 0.003 c	0.013 ± 0.004 a	0.092 ± 0.004 c	0.063 ± 0.003 b
**Terpenes**							
limonene	citrus[2*]	0.146 ± 0.004 A	0.581 ± 0.014 B	0.068 ± 0.001 a	0.440 ± 0.021 c	0.650 ± 0.021 d	0.295 ± 0.009 b
3-pinanone	cedar camphoreous[1*]	0.110 ± 0.002 A	0.285 ± 0.009 B	0.408 ± 0.016 d	0.043 ± 0.002 a	0.133 ± 0.036 b	0.206 ± 0.016 c
p-Menth-1-en-4-ol	Pepper, woody, earth, musty, sweet[1*]	0.010 ± 0.004 A	0.022 ± 0.011 B	0.001 ± 0.000 a	nd	0.051 ± 0.002 c	0.012 ± 0.003 b
cis-*p*-Menth-8-en-2-one	herbal warm[1*]	nd	0.116±0.007	nd	nd	0.226 ± 0.013 b	0.005 ± 0.000 a
trans-*p*-Menth-8-en-2-one	warm herbal[1*]	nd	0.069 ± 0.005	nd	nd	0.139 ± 0.008	nd
p-Menth-6,8-dien-2-one	minty licorice[1*]	0.047 ± 0.002 A	0.392 ± 0.021 B	0.149 ± 0.009 b	0.006 ± 0.000 a	0.694 ± 0.040 c	0.030 ± 0.006 a

				**Others**			
acetic acid	acidic[2*]	1.497 ± 0.273 A	4.859 ± 0.115 B	1.149 ± 0.085 a	4.381 ± 0.214 c	2.099 ± 0.319 b	5.083 ± 0.146 d
dill ether	at 10.00% in dipropylene glycol. dill[1*]	0.033 ± 0.002 A	0.230 ± 0.011 B	0.017 ± 0.002 a	0.039 ± 0.002 a	0.350 ± 0.020 b	0.119 ± 0.075 a
acetophenone	almond, flower[4*]	0.020 ± 0.004 A	0.073 ± 0.003 B	0.018 ± 0.003 a	0.085 ± 0.004 c	0.048 ± 0.017 b	0.035 ± 0.003 b
5,6-dihydro-2H-pyran-2-one		3.082 ± 0.067 A	3.804 ± 0.103 A	2.117 ± 0.105 a	0.819 ± 0.139 a	6.321 ± 0.754 c	4.514 ± 0.750 b
methyl eugenol	sweet, fresh, warm spicy, clove carnation cinnamon[1*]	0.041 ± 0.002 A	0.047 ± 0.002 B	0.066 ± 0.004 c	0.062 ± 0.003 c	0.032 ± 0.002 b	0.016 ± 0.001 a

nd – not detected. Different letters in rows for the given attribute denote statistically significant differences at $p<0.05$. Capital letters denote significance as affected by the sucrose saturation and lowercase letters denote significance as affected by the preservation method. Superscript numbers with an asterisk denote source of the odor description for the respective volatile compound: 1* - [18]; 2*- [19]; 3* - [20]; 4* - [21].
